# Clinical course of new-onset Crohn’s disease in children and adolescents in dependency of age, initial location, initial severity level and therapy over the period 2000–2014 based on the Saxon Pediatric IBD-Registry in Germany

**DOI:** 10.1371/journal.pone.0287860

**Published:** 2023-06-29

**Authors:** Fan Dong, Ivana Kern, Jens Weidner, Kathleen Kügler, Ulrike Rothe, Makan Amin, Martin W. Laaß, Gunter Flemming, Ulf Winkler, Thomas Richter, Joachim Kugler, Ulf Manuwald

**Affiliations:** 1 Health Sciences/Public Health, Institute and Policlinic for Occupational and Social Medicine, Faculty of Medicine “Carl Gustav Carus”, TU Dresden, Dresden, Germany; 2 Center for Medical Informatics, Institute for Medical Informatics and Biometry, Faculty of Medicine “Carl Gustav Carus”, TU Dresden, Dresden, Germany; 3 General practice A. Wolf, Freital, Germany; 4 GWT of the TU Dresden, Dresden, Germany; 5 Department for Trauma Surgery and Orthopedics, Hospital “Park-Klinik Weissensee”, Berlin, Germany; 6 University Hospital for Children and Adolescents, Faculty of Medicine “Carl Gustav Carus”, TU Dresden, Dresden, Germany; 7 Department of Gastroenterology, Hospital for Children and Adolescents, University of Leipzig, Leipzig, Germany; 8 Clinic for Children and Adolescents, Hospital Bautzen, Oberlausitz-Kliniken gGmbH, Bautzen, Germany; 9 Clinic St. Georg, Leipzig, Germany; University of Lille, FRANCE

## Abstract

**Objective:**

In Saxony, the incidence of Crohn’s disease (CD) in children and adolescents increased significantly from 3.3 per 100,000 person-years in 2000 to 5.1 in 2014. The aim of this study was to describe the initial characteristics and the clinical course of CD in children and adolescents and to identify drug treatment options associated with an advantage for a mild course or remission.

**Methods:**

Clinical data were collected from patients who suffered from inflammatory bowel disease (IBD) and were recruited in the Saxon Pediatric IBD-Registry. All children newly diagnosed with CD in this registry in Saxony between 2000 and 2014 were included in this registry study. Characteristics such as age, disease location and extra-intestinal manifestations at diagnosis were accessed. The severity level of the disease at diagnosis as well as at follow-up were analysed by PCDAI index. Patients were divided into 3 groups according to length of follow-up: 1–3 years, 4–6 years and 7–9 years after diagnosis. A logistic regression model was conducted to examine which baseline parameters are associated with disease progression.

**Results:**

There were 338 children and adolescents with CD included in this registry study. At diagnosis, the median age of patients was 12.0 (0.7–14.9), 61.5% (n = 208) of the patients were male. The most common disease location observed in pediatric CD patients was the L3 (55%, n = 176). Patients aged 10–14 years were significantly more likely to present an L2 than patients aged 0–4 years (80.3%, n = 53 vs. 19.7%, n = 13, p = 0.01). During the follow-up, data from 71.3% (n = 241) othe patients were available. Disease activity measured by PCDAI decreased in 47.7% (n = 115) of the patients, 40.7% (n = 98) of the patients were stable and increased in 11.6% (n = 28) of the patients. Patients with intermediate/severe disease at onset were more likely to have an active disease at the end of follow up, too (p = 0.00). Logistic regression analysis of the initial characteristics showed that the age at diagnosis, gender, initial location and initial extra-intestinal manifestation are not associated with the progression of the disease (p>0.05). Furthermore, drug treatment options could be identified from our data, which are associated with benefits for a milder course or remission.

**Conclusion:**

From 2000 to 2014, the health status of most pediatric patients with CD had improved or remained stable. Initial characteristics including age at diagnosis, initial localization and initial extra-intestinal manifestation are not associated with the progression of the disease, only the initial activity by PCDAI.

## Introduction

Crohn’s disease (CD), a chronic inflammation of the digestive tract from the mouth to the anus with mostly manifestation in the terminal ileum and colon, is part of a group of diseases known as inflammatory bowel diseases (IBD). Crohn, Ginzburg and Oppenheimer provided the first description of terminal ileitis in 1932 [1].

Pediatric CD has garnered much attention recently due to its fast-increasing incidence. The incidence of CD in children and adolescents per 100,000 person-years in the years 2000 onwards ranged from 2.1–15.3 in Western Europe, from 1.3–15.3 in United Stated of America, 0–15.3 in Eastern Europe, but only 3.5–5.9 in Oceania and 0.3–2.8 in Eastern Asia [2]. In Saxony the age-standardized incidence rate of pediatric CD was 4.8 per 100,000 person-years (PY) for the period 2000 to 2014 [3].

Clinical course of pediatric CD was associated with initial characteristics such as age at diagnosis, location and initial disease severity [4–10]. So far, prediction of the clinical course of pediatric CD using initial clinical features has not yet been achieved. A systematic study concluded that there were no predictors for future disease severity level in pediatric IBD [11].

The aim of our study was to examine the clinical course depending on characteristics such as age at diagnosis, initial location and initial disease severity level. In addition, we investigated the impact of different treatment options on inflammatory activity using the data of the Saxon Pediatric IBD Registry between 2000 and 2014.

## Methods

Data from the Saxon Pediatric IBD-Registry were used in this study to evaluate the progression of disease. Saxony is a federal state in Germany with an area of 18,415 square kilometres and a population between 4.43 and 4.06 million from 2000 to 2014 [12].

### Data source

The Saxon Pediatric IBD-Registry was established in 2000.This registry received information from all 31 pediatric hospitals that reported this data. More detailed information can be found in Kern et al. 2021 [13]. Patients in the registry aged 0–14 years and newly diagnosed with CD between 2000 and 2014 were eligible for this follow-up study. Individual follow-up time is the period from the first report (diagnosis) to the last report form of a patient. Patients with missing data and those without available follow-up results for at least one year following diagnosis were excluded. Patients were divided by age at diagnosis. The age groups were as follows: 0–4 years, 5–9 years and 10–14 years.

The Saxon Pediatric IBD Registry’s completeness was estimated to be 96.7% [14].

### Definition and classification

#### Diagnostic of pediatric CD

Pediatric CD was diagnosed using the Porto criteria. The diagnosis based on clinical signs and symptoms, endoscopy, histology and radiology [15].

#### Initial disease location

The disease location at diagnosis is regarded as the initial disease location. It was determined using the Paris classification criteria [16]. The intestinal involvement in CD is divided into L1,L2,L3,L4a and 4b. Upper digestive tract(UGI), meaning L4a and L4b,can be combined with other subgroups. The initial location of the disease was calculated as the location up to 500 days after the date of diagnosis.

#### Clinical course

The Pediatric Crohn’s Disease Severity Index (PCDAI) was used to assess the disease severity. The PCDAI score includes assessment of 3 categories: clinical symptoms, physical examination and laboratory results. Generally, the score is arranged from 0 to 100. These are cut points: remission <10, mild 10–30, intermediate/severe 30–100 [17].

The score was calculated at diagnosis as well as 1–3 years, 4–6 years and 7–9 years after diagnosis If many visits were made throughout these time periods, the registration form with the highest severity level was chosen for assessment. Besides, if there were several highest level, the last year was utilised for assessment. When disease severity level is unavailable at the time of diagnosis, disease severity occurring within 1.5 months after the diagnosis is considered as initial.

#### Treatment options

To investigate a relationship between therapy and inflammatory activity, we performed additional analyses on different treatment options according to the "ECCO/ESPGHAN Consented Guidelines on Drug Treatment of Pediatric Crohn’s Disease" [18]. We investigated drug monotherapies as well as therapy combinations.

### Statistical analysis

SPSS 26.0 and the package "epitools" version 0.5–10.1 of R software version 4.2.1 were used to perform the statistical analyses. Independent variables were age at diagnosis (0–4, 5–9, 10–14), lower digestive tract (L1, L2, L3), UGI (yes or none), extra-intestinal manifestations (yes or none). The dependent variable was the development of the disease (improved, deteriorated). Data for the continuous variable such as age were displayed as median. Categorical data were presented as frequencies and percentages. A comparison of initial characteristics parameters across 3 age groups was performed using chi-square test or t-test. A probability of error of 5% was defined a priori. At the end of follow-up, patients were classified into one of 2 groups according to the clinical course: improvement or deterioration. Only patients with a change in categories "remission, mild, intermediate/severe" at diagnosis and at the end of follow-up, (or in the 3-year time periods), were included in the model. The values of baseline variables for both groups were calculated and the significance of the difference between both groups was tested using t-test or chi square test as appropriate. A binary logistic regression model was conducted to examine which baseline parameters are associated with disease progression. Odds ratios with 95% confidence interval (CI) from multivariate logistic regression models were used to measure the effect of the initial clinical parameter on disease progression in patients with improvement or deterioration. McNemar-Test was used to determine if there are differences in disease severity at the end of the follow-up between patients in remission at diagnosis and those with an active disease. To establish associations on the impact of drug treatment options on inflammatory activity, odds ratios were calculated generically for the pediatric cohort of the registry with 95% CI.

### Ethics

The study was approved by the Ethical of the University of Leipzig (Reg. No. 033/2000).

Prior to data collection, the legal guardian signed written informed consent forms.

## Results

### Baseline characteristics

From 2000 to 2014, a total of 338 patients diagnosed with CD were identified. The median age at diagnosis of them was 12.0 years (range 0.7–14.9), 61.5% (n = 208) were male. The baseline characteristics of 338 patients are highlighted in S1 Table.

At time of diagnosis, most of the patients with newly diagnosed CD were in the age group between 10–14 years of age. More than half of the patients in each age group were male. 71.9% of the patients (n = 243, 65.5% male) were 10–14 years of age, 19.5% (n = 66, 51.5% male) were 5–9 years old and 8.6% (n = 29, 63.8% male) were 0–4 years old.

**Fig 1:**
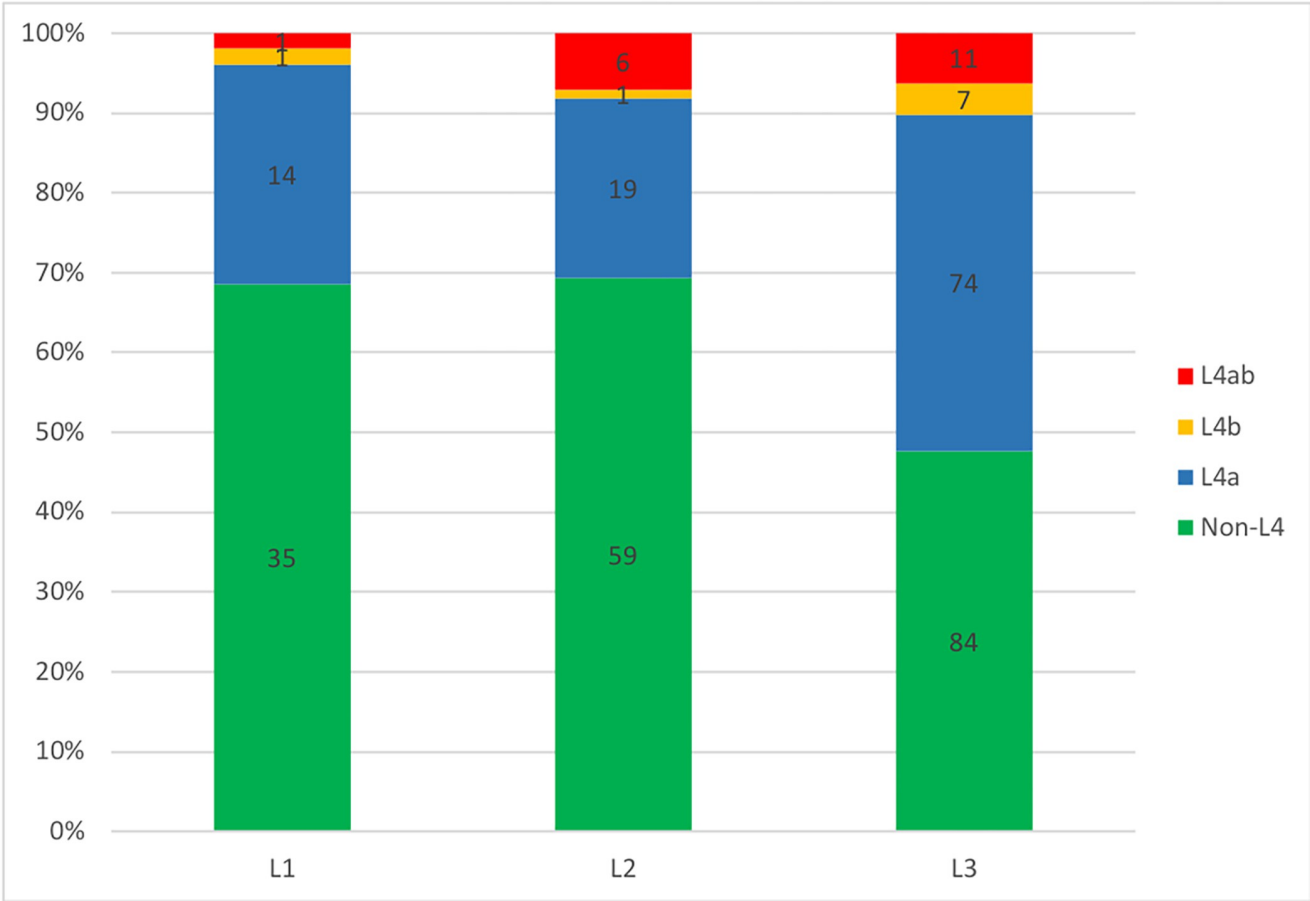
UGI involvement and concurrent disease location. L1: distal 1/3 ileum±limited cecal disease; L2: colonic; L3: ileocolonic; L4a: upper disease proximal to Ligament of Treitz; L4b: upper disease distal to ligament of Treitz and proximal to distal 1/3 ileum.

The initial location was observed for 320/338 patients, it was classified according to the Paris classification. The most frequently location in pediatric patients with CD was L3 with 55% (n = 176), followed by UGI involvement with 44.4% (n = 142), L2 with 26.6% (n = 85) and L1 with 15.9% (n = 51). 41.9% (n = 134) of the patients had concomitant disease of UGI with one of L1, L2 or L3, whereas barely 2.5% (n = 8) of the patients had isolated UGI disease (Fig 1). UGI (n = 142) = L1/UGI (16) + L2/UGI (26) + L3/UGI (92) + isolated UGI (8).

In general, there were significant differences in the distribution of disease location among the 3 age groups (p = 0.03). The frequency of L2 differed significantly between patients aged 10–14 and those aged 0–4. Patients aged 10–14 years were more likely than those aged 0–4 years to present on L2 (80.3%, n = 53 vs 19.7%, n = 13, p = 0.01). This suggests that phenotypic features vary in adolescents aged 10–14 years and younger children. There were no significant variations between age groups for L1, L3 and UGI.

The differences in disease location across gender groups were not statistically significant (p>0.05).

Initial extra-intestinal manifestations were not found in 2/3 of the patients (62.7%, n = 190). These extra-intestinal manifestations, such as knee joint manifestation, skin manifestations, hepatobiliary and ocular manifestations and spine manifestation were observed in 113/303 patients at diagnosis. Knee joint manifestations (18.5%, n = 56) were most often reported, followed by skin (10.6%, n = 32), liver (4.6%, n = 14), and eye (3.6%, n = 11). Primary sclerosing cholangitis (PSC) was found in 3 cases and the spine was found in 2.

No significant differences were found in the distribution of extra-intestinal manifestations among the 3 age groups (p>0.05) or between gender groups (p>0.05).

### Clinical course of CD

#### Disease severity level at diagnosis

At diagnosis, PCDAI was collected for 308/338 patients. Among them, 37.1% (n = 114) were in remission, whereas a considerable number of patients (62.9%, n = 194) had an active disease, which includes half (n = 154) of patients with mild disease and 13% (n = 40) with intermediate to severe disease.

**Fig 2:**
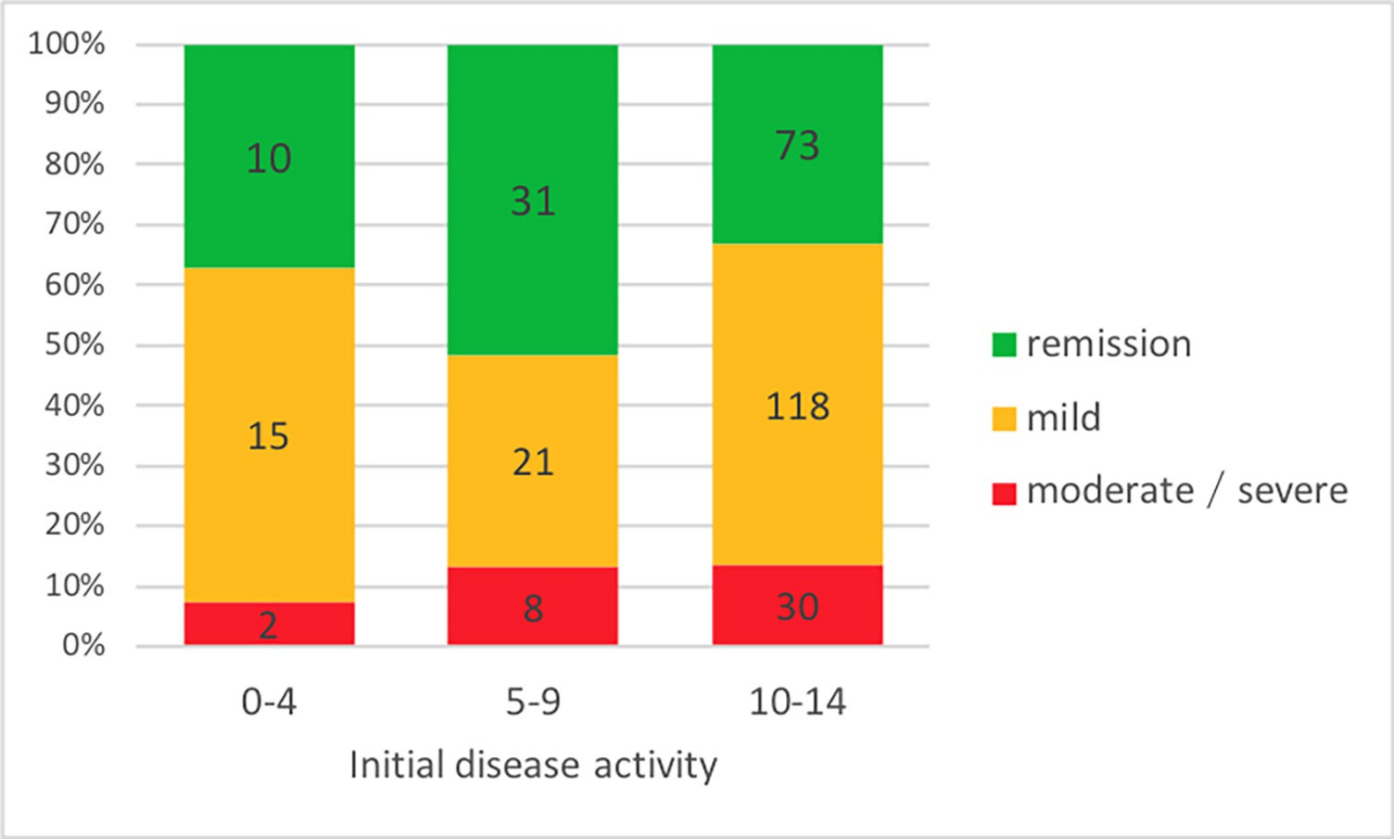
Age distribution of initial disease severity (PCDAI) in pediatric CD.

Distribution of active diseases differed significantly for patients categorized by age at diagnosis (Fig 2). The disease was active in more than 2/3 of the patients (67.0%, n = 148) in the age group 10–14 years, while less than half of the patients (48.3%, n = 29) aged 5–9 years had an active disease (p = 0.01).

There was no significant difference between genders for distribution of disease severity at diagnosis (p>0.05).

#### Disease severity level at follow-up

Data on disease severity during follow-up were available for 71.3% (n = 241) of CD patients.

**Fig 3:**
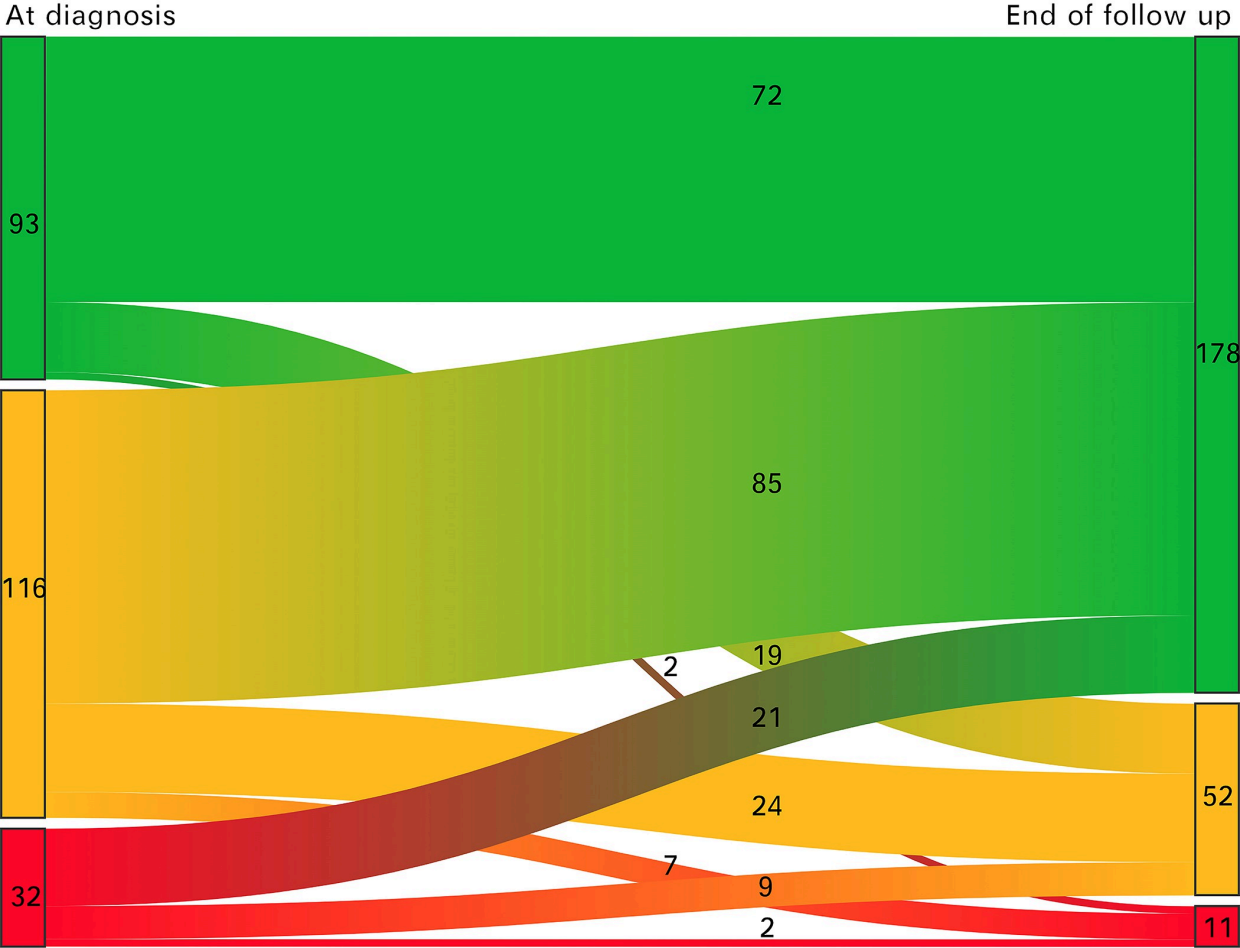
Observation of all patients from diagnosis to the end of follow-up. Green = Remission; Orange = Mild; Red = Intermediate/sever.

The movement of the patient’s disease severity from diagnosis to the end of follow-up is presented in Fig 3.

Among all 241 patients, the health status of 47.7% (n = 115) of the patients improved, 40.7% (n = 98) of the patients were stable, and 11.6% (n = 28) of the patients worsened.

In total, the rates of remission in the patients increased from 37.0% (n = 114) at diagnosis to 63.2% (n = 196) at the end of follow-up (p = 0.00).

In all 3 groups with different disease severity at diagnosis, most of the patients were in remission, followed by mild disease and intermediate/severe disease at the end of follow-up.

Data regarding disease severity at 1–3 years (n = 176), 4–6 years (n = 134) and 7–9 (n = 36) years after diagnosis were available for analysis.

**Fig 4:**
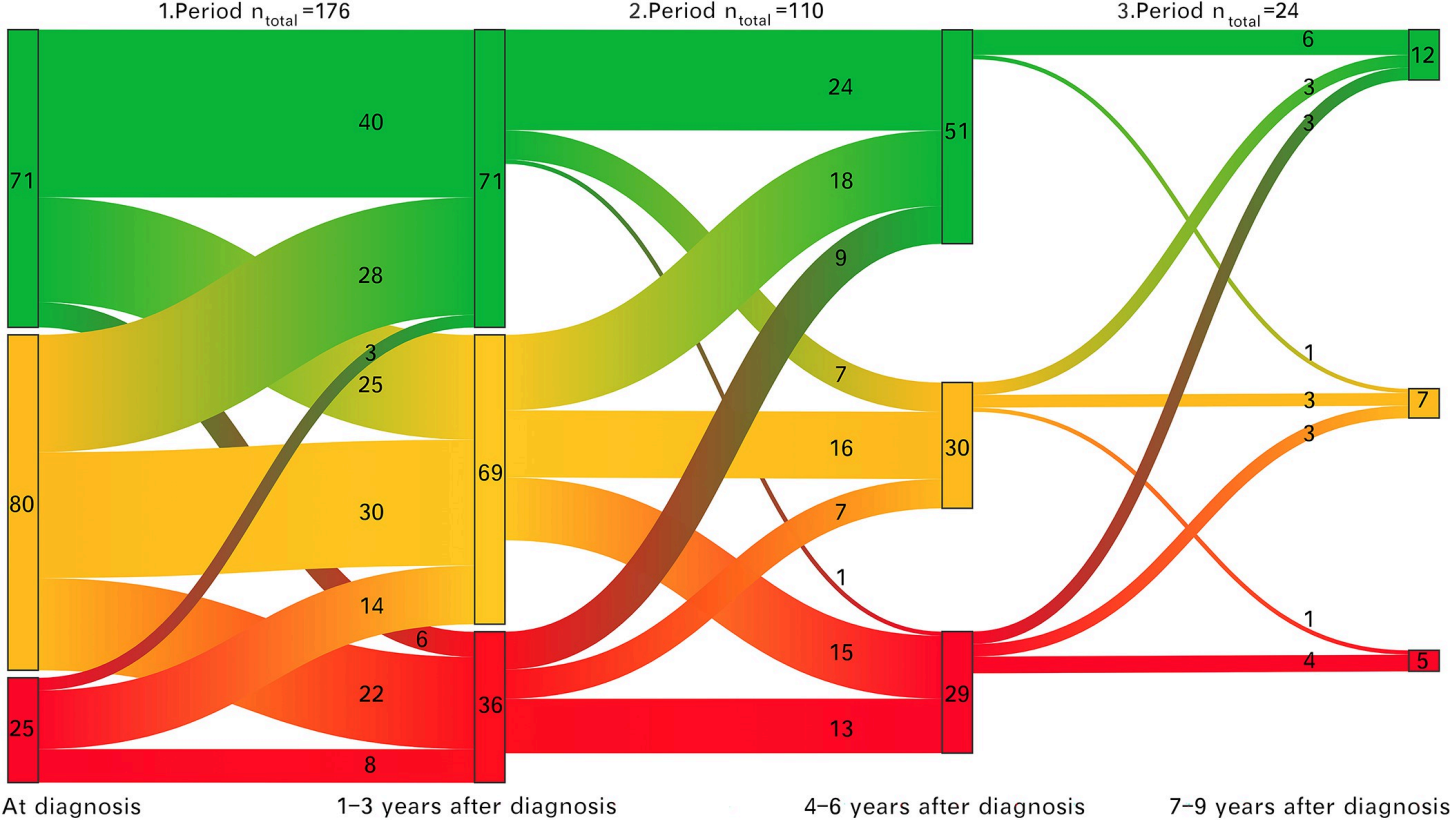
Disease progression over the 3 time periods. Green = Remission; Orange = Mild; Red = Intermediate/severe.

Among these patients, the distribution of disease severity remained stable during the periods 1–3 years, 4–6 years and 7–9 years after diagnosis. The proportion of patients with disease remission was 40.3% (n = 71), 47.8% (n = 65) and 44.4% (n = 16) during the periods as above, respectively (p = 0.36). The proportion of patients with mild disease was 39.2% (n = 69), 27.2% (n = 37) and 38.9% (n = 14) during these 3 periods, respectively (p = 0.06). The proportion of patients with intermediate/severe disease was 20.5% (n = 36), 23.5% (n = 32) and 16.7% (n = 6) during the 3 periods, respectively (p = 0.58). Disease progression over the 3 time periods is indicated in Fig 4.

Patients remaining stable in disease severity over the 3 time periods were the most frequent (>40%). From diagnosis to 1–3 years after diagnosis, 25.6% (n = 45) of the patients had lower disease activity, 44.3% (n = 78) of the patients were stable, and 30.1% (n = 53) patients had higher disease activity. In 66 patients with data for 1–3 years after diagnosis, data for 4–6 years after diagnosis were not available. Among the remaining 110 patients, the disease activity decreased in 30.9% (n = 34) of the patients, 48.3% (n = 53) of the patients were stable and the disease activity increased in 20.9% (n = 23) of the patients. Data for 7–9 years after diagnosis were not available in 86 of the patients who were included in time period 4–6 years after diagnosis. Among the remaining 24 patients, at 37.5% (n = 9) of the patients the health status improved, 54.2% (n = 13) of the patients remained stable and at 20.8% (n = 5) of the patients the health status worsened.

#### Prediction of clinical course of CD

The effect of variables such as age at diagnosis, sex, initial location including distal location and UGI, initial extra-intestinal manifestations and initial disease severity on the course of the disease was examined.

The McNemar-Test demonstrated a statistically significant difference in remission rates at the end of follow-up between patients in remission at diagnosis and those who were not (59.6%, n = 106 vs 40.4%, n = 72, p = 0.00). This shows that individuals in remission at the time of diagnosis had a better clinical course than those who were not in remission at the time of diagnosis.

In order to examine which baseline parameters are associated with disease progression, 143/241 patients were divided into 2 groups, the improvement group, which represents patients who had lower disease severity at the end of follow-up than at diagnosis, and the deterioration group, which represents patients who had higher disease severity at the end of follow-up. The characteristics of patients at baseline between the 2 groups were compared. There was no significant difference in the distribution of initial characteristics between the 2 groups (p>0.05).

Univariate logistic analysis and multivariate logistic regression analysis were conducted to assess the effect of 4 variables on clinical course. The predictor variables were age, distal disease location, UGI, and extra-intestinal manifestations at diagnosis. The Hosmer-Lemeshow-Test indicated a good match (p = 0.211). Logistic regression analysis revealed that there was no statistically significant association between disease severity at diagnosis and disease progression (p>0.05) (Table 1).

**Table 1:** Logistic regression regarding improved vs. deteriorated cases in relation to the beginning of the disease.

Parameter	Univariate analysis (N = 143)		Multivariate analysis (N = 143)	
	P values	OR (95%CL)	P values	OR (95%CI)
**Age at diagnosis (N = 143)**				
0–4 (n = 12)	0.58	Reference	0.60	Reference
5–9 (n = 26)	0.90	1.11 (0.13–3.78)	0.7	0.76 (0.14–4.04)
10–14 (n = 105)	0.32	0.60 (0.20–1.67)	0.33	0.59 (0.20–1.71)
**Location (n = 138)**				
L1 (n = 19)	0.94	Reference	0.83	Reference
L2 (n = 34)	0.92	0.94 (0.28–3.19)	0.75	0.81 (0.23–2.87)
L3 (n = 85)	0.77	1.17 (0.42–3.27)	0.67	1.28 (0.41–4.01)
**UGI (N = 138)**	0.81	0.90 (0.38–2.12)	0.89	0.94 (0.38–2.32)
**EIMs (N = 138)**	0.89	0.94 (0.40–2.24)	0.97	1.02 (0.42–2.48)

R^2^ = 0.021; L: Lower digestive tract; L1: distal 1/3 ileum±limited cecal disease; L2: colonic; L3: ileocolonic; UGI: Upper gastrointestinal tract = L4a (upper disease proximal to Ligament of Treitz), L4b (upper disease distal to ligament of Treitz and proximal to distal 1/3 ileum) and L4ab; EIMs: Extra-intestinal manifestations; N = group of patients studied; n = number of cases

#### Impact of therapy on inflammatory activity

**Fig 5:**
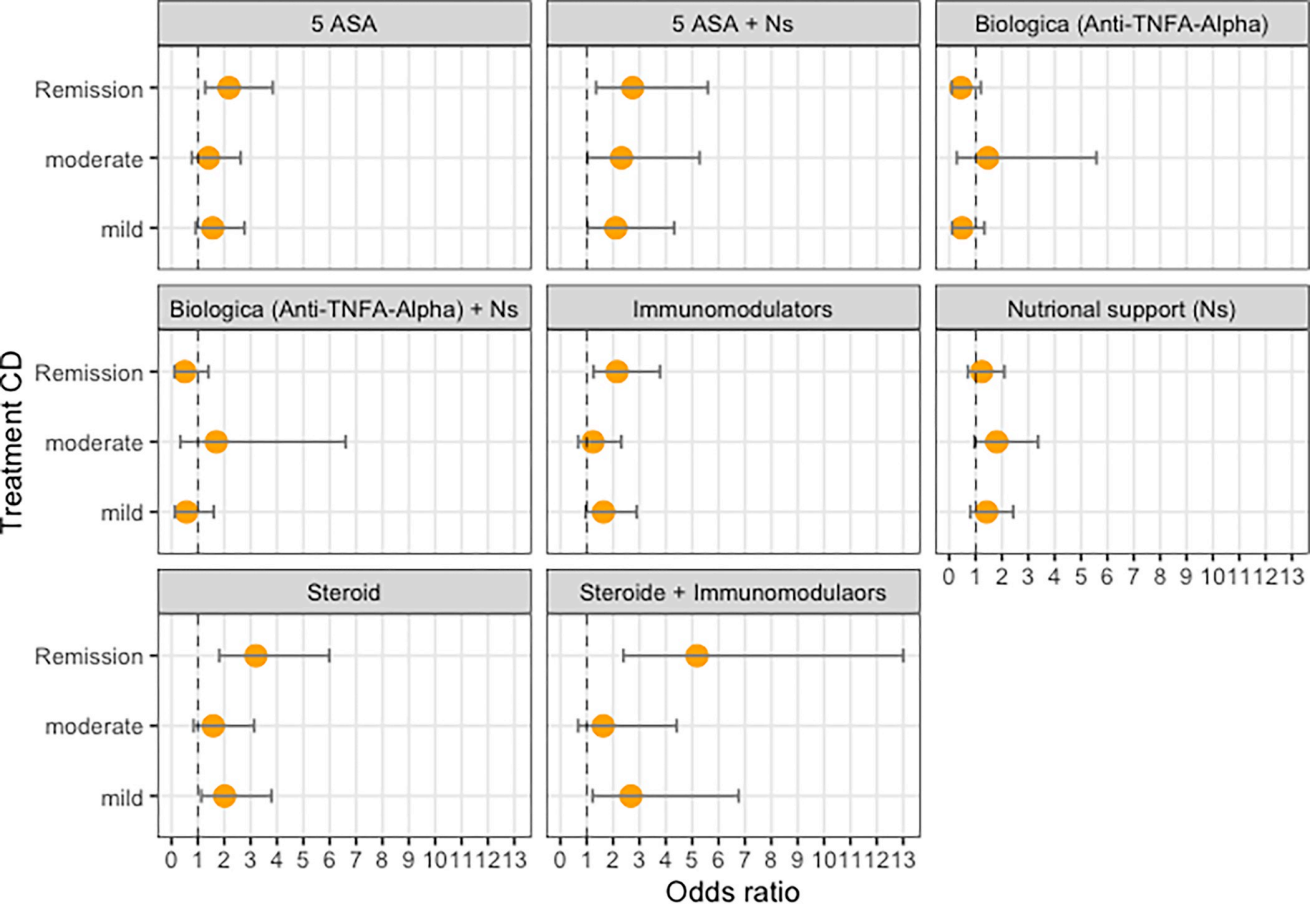
Association between drug therapy options and inflammatory activity, reference “severe”.

Our analysis showed that monotherapy with 5-ASA therapy with steroids, or immunomodulators had a positive effect on reducing inflammatory activity (S2 Table and Fig 5). In addition, therapeutic combinations of 5-ASA and nutritional support and steroids and immunomodulators were also associated with significantly lower inflammatory activity. However, no statistically significant association was found between therapy with biologics (anti-TNFA-alpha) and reduced inflammatory activity, whether administered alone or in combination with nutritional therapy.

## Discussion

This registry study adds new information on the initial characteristics and clinical course of CD in children and adolescents in Saxony.

Consistent with previous studies, this research found that CD was more prevalent in patients aged 10–14 years (79.1%.n = 243) [19–21] than in patients aged 0–4 years (8.6% n = 29). Previous research showed a slightly higher proportion of individuals under 5 years old at diagnosis (9.9% -11%) [5, 6].

In this study, the most frequent location in pediatric CD patients was L3 (55%, n = 176). Similar percentages (52.6%-61%) were also reported by many previous studies [19, 21–23]. The UGI was not frequently involved, only 44% (n = 142) of patients presented with UGI involvement. Proportion of patients with UGI involvement observed in our study are below those observed by Kim et al. [24] and Crocco et al. [25] (74.4%,53.3%, respectively). This inconsistency may be due to genetic variations [19, 21–23]. Patients aged 10–14 years had significantly higher L2 involvement than younger children aged 0–4 years (80.3%, n = 53 vs 19.7%, n = 13, p = 0.01). This outcome is contrary to that of Gupta et al. [5] who found that there were no disparities in disease location between patients in different age groups. Gupta et al. [5] concentrated on the colon and small bowel only, whereas the description of location in our study using Pairs classification was more detailed.

A significant trend toward reduction in disease activity has been observed at most of the patients (47.7%, n = 115) during the 15 years of follow-up. This finding is consistent with earlier observations, which found that disease severity at the end of follow-up was reduced compared to severity at diagnosis [19–21]. Theses suggest that the clinical course of patients with pediatric CD has changed, which is not as serious as previously reported [26].

Our data demonstrate that age and disease location are not associated with the disease severity of clinical course of CD in children and adolescents (p>0.05). It is well established that older children had a higher probability of bowel surgery within 5 years of diagnosis [11]. However, the risk of surgery cannot be used as a primary assessment of disease severity, because surgery is not always harmful and can prolong the period of clinical remission [27]. Moreover, we did not observe that individuals with extensive intestinal involvement experience a severe clinical course (p>0.05). This finding was also reported by Greef et al. [21] who found that L1 and L3 are not associated with disease severity in multiple logistic regression. Crocco et al. [25] reported that there was no difference in mean PCDAI scores at the end of follow-up between patients with and without upper gastrointestinal involvement.

Notably, at the end of observation, there was a statistically significant difference in remission rates between patients who were in remission and were not in remission at diagnosis (59.6%, n = 106 vs 40.4%, n = 72 p = 0.00). Moreover, previous studies have indicated that patients with mild disease had better outcomes than patients with intermediate to severe disease during the follow-up [7]. In general, therefore, it seems that disease activity at time of diagnosis is associated with disease outcome [18, 28]. For maintenance of remission, one clinical trial showed a twofold reduction in the risk of relapse, but overall, there is insufficient evidence to support maintenance therapy with 5-ASA [18, 29]. However, our results suggest that therapy with 5ASA is associated with a benefit in favor of remission. We would, however, need to perform further and deeper analyses with subgroups to make a robust conclusion. Based on our results, we can follow the recommendation of Ruemmele et al. (2014) that treatment with 5ASA can be used to induce remission in children with mild inflammatory activity but should be considered as adjuvant therapy. It also appears from our results that adjunctive nutritional support can support therapy with 5ASA. Our further results show that a therapy with steroids, immunomodulators or a combination therapy of steroids and immunomodulators are associated with advantages for a remission or milder course. Therefore, our results are largely consistent with the recommendations "Consensus guidelines of ECCO/ESPGHAN on the medical management of pediatric Crohn’s disease" [18, 30]. However, deeper investigation of our data is needed to substantiate the benefits of individual treatment options.

### Strengths and limitations

The current study is the first population based long-term registry study in Germany to explore the initial characteristics and clinical course of CD in children and adolescents. The completeness of the database was estimated to be 97% [7]. Therefore, the findings of this study provide persuasive evidence for the specificity of CD clinical features in children and adolescents and imply that the clinical course of CD in children and adolescents appears to shift with a propensity toward unaggressive. We are unable to identify initial clinical variables associated with the severity of clinical course, but initial disease activity in remission could be used a reference indicator for a stable clinical course.

However, significant limitations should to be noted. First, before application of the Porto criteria released in 2005, the Lenard-Jones criteria were recommended in Saxony [31]. The initial registry form including diagnostic information was updated for over 15 years and difficult cases were reviewed at the registry meetings. Therefore, changes in diagnostic criteria do not affect the correct classification of IBD. Also, although the influence of initial clinical features on the clinical course has been examined, the effect of therapy and environmental factors on the clinical course was not considered. But we conclude from our results–no adherence from age and location–that clinical course could be related to the treatment in Saxony. Clinical course could be related to new treatments and changes in environmental factors [19–21]. Therefore, further studies ought to take into account these variables.

## Conclusion

From 2000 to 2014, most of the patients with pediatric CD had a stable course of the disease or achieved improvement of health status in the long term. It also proves a therapeutic success. Only a minority of patients developed a more aggressive form of disease at the end of follow-up than at the beginning. Patients with intermediate/severe disease at diagnosis are more likely to present also an active disease at the end of follow-up.

## Supporting information

S1 Table Baseline characteristics and comparison of variables among the 3 age groups. UGI: Upper gastrointestinal tract; L1: distal 1/3 ileum±limited cecal disease; L2: colonic; L3: ileocolonic; L4a: upper disease proximal to Ligament of Treitz; L4b: upper disease distal to ligament of Treitz and proximal to distal 1/3 ileum; PSC: primary sclerosing cholangitis; EIMs: extra-intestinal manifestations.
(DOCX)

S2 Table Impact of Therapies on inflammatory activity, reference “severe”. (DOCX)
