# Supplementary material for: Clinical course of new-onset Crohn’s disease in children and adolescents in dependency of age, initial location, initial severity level and therapy over the period 2000–2014 based on the Saxon Pediatric IBD-Registry in Germany
Source: PLoS One. 2023 Jun 29;18(6):e0287860. doi: 10.1371/journal.pone.0287860 (PMC10309614; doi:10.1371/journal.pone.0287860)
Supplement: S1 Table — UGI: Upper gastrointestinal tract; L1: distal 1/3 ileum±limited cecal disease; L2: colonic; L3: ileocolonic; L4a: upper disease proximal to Ligament of Treitz; L4b: upper disease distal to ligament of Treitz and proximal to distal 1/3 ileum; PSC: primary sclerosing cholangitis; EIMs: extra-intestinal manifestations. (DOCX) [file pone.0287860.s001.docx]

**S1 Table.** Baseline characteristics and comparison of variables among the 3 age groups

|  | Total  N=338 (100%) | | Age 0-4 yr  N=29(8.6%) | | Age 5-9 yr  N=66(19.5%) | | Age 10-14 yr  N=243(79.1%) | | P  Values |
| --- | --- | --- | --- | --- | --- | --- | --- | --- | --- |
| Sex |  |  |  |  |  |  |  |  |  |
| Male | 208 | 61.5% | 19 | 65.5% | 34 | 51.5% | 155 | 63.8% | 0.18 |
| Female | 130 | 38.5% | 10 | 34.5% | 32 | 48.5% | 88 | 36.3% | - |
| Location (n=320) |  |  |  |  |  |  |  |  |  |
| UGI only | 8 | 2.5% | 1 | 3.6% | 3 | 5.0% | 4 | 1.7% | 0.03 |
| L1 | 51 | 15.9% | 1 | 3.6% | 6 | 10.0% | 44 | 19.0% | - |
| L2 | 85 | 26.6% | 13 | 46.4% | 19 | 31.7% | 53 | 22.8% | - |
| L3 | 176 | 55% | 13 | 46.4% | 32 | 53.3% | 131 | 56.3% | - |
| UGI |  |  |  |  |  |  |  |  |  |
| None | 178 | 55.6% | 19 | 67.9% | 28 | 46.7% | 131 | 56.3% | 0.55 |
| L4a | 113 | 35.3% | 7 | 25.0% | 25 | 41.7% | 81 | 34.9% | - |
| L4b | 10 | 3.1% | 2 | 7.1% | 3 | 5.0% | 7 | 3.0% | - |
| L4ab | 19 | 5.9% | 4 | 14.3% | 13 | 21.7% | 2 | 0.9% | - |
| Subgroup |  |  |  |  |  |  |  |  |  |
| L4+L1 | 16 | 5% | 0 | - | 1 | 1.7% | 15 | 6.5% | 0.14 |
| L4+L2 | 26 | 8.1% | 1 | 3.6% | 9 | 15.0% | 16 | 6.9% | 0.69 |
| L4+L3 | 92 | 28.8% | 7 | 25.0% | 20 | 33.3% | 66 | 28.4% | 0.80 |
| EIMs  (n=303) |  |  |  |  |  |  |  |  |  |
| Eyes | 11 | 3.6% | 0 | - | 3 | 4.7% | 8 | 3.8% | 0.69 |
| Skin | 32 | 10.6% | 1 | 3.8% | 5 | 7.8% | 26 | 12.2% | 0.37 |
| Liver | 14 | 4.6% | 3 | 11.5% | 4 | 6.3% | 7 | 3.3% | 0.09 |
| Joints | 56 | 18.5% | 3 | 11.5% | 12 | 18.8% | 41 | 19.2% | 0.72 |
| PSC | 3 | 1% | 0 | - | 0 | - | 3 | 1.4% | 1.00 |
| Spinal-column | 2 | 0.7% | 0 | - | 1 | 1.6% | 1 | 0.5% | 0.51 |
| Others | 33 | 10.9% | 0 | - | 6 | 9.4% | 27 | 12.7% | - |
| None | 190 | 62.7% | 19 | 73.1% | 42 | 65.5% | 129 | 60.6% | 0.33 |

*Legend: UGI: Upper gastrointestinal tract; L1: distal 1/3 ileum*$\pm$*limited cecal disease; L2: colonic; L3: ileocolonic; L4a: upper disease proximal to Ligament of Treitz;*

*L4b: upper disease distal to ligament of Treitz and proximal to distal 1/3 ileum; PSC: primary sclerosing cholangitis; EIMs: extra-intestinal manifestations*
