# Supplementary material for: Clinical course of new-onset Crohn’s disease in children and adolescents in dependency of age, initial location, initial severity level and therapy over the period 2000–2014 based on the Saxon Pediatric IBD-Registry in Germany
Source: PLoS One. 2023 Jun 29;18(6):e0287860. doi: 10.1371/journal.pone.0287860 (PMC10309614; doi:10.1371/journal.pone.0287860)
Supplement: S2 Table — (DOCX) [file pone.0287860.s002.docx]

**S2 Table**: Impact of Therapies on inflammatory activity, Reference “severe”

| Treatment | Disease activity | OR | lower | upper | p-val |
| --- | --- | --- | --- | --- | --- |
| 5 ASA | Remission | 2,18* | 1,28 | 3,84 | 0,00 |
|  | mild | 1,56 | 0,91 | 2,77 | 0,11 |
|  | moderate | 1,40 | 0,77 | 2,62 | 0,26 |
| Steroid | Remission | 3,19* | 1,81 | 5,98 | 0,00 |
|  | mild | 2,01* | 1,13 | 3,79 | 0,00 |
|  | moderate | 1,58 | 0,83 | 3,13 | 0,16 |
| Immunomodulators | Remission | 2,14* | 1,26 | 3,78 | 0,00 |
|  | mild | 1,63 | 0,95 | 2,89 | 0,07 |
|  | moderate | 1,23 | 0,67 | 2,31 | 0,49 |
| Biologica (Anti-TNFA-Alpha) | Remission | 0,43 | 0,10 | 1,20 | 0,13 |
|  | mild | 0,48 | 0,11 | 1,33 | 0,18 |
|  | moderate | 1,45 | 0,28 | 5,58 | 0,62 |
| Nutrional support (Ns) | Remission | 1,23 | 0,70 | 2,09 | 0,45 |
|  | mild | 1,42 | 0,80 | 2,43 | 0,21 |
|  | moderate | 1,80 | 0,95 | 3,37 | 0,06 |
| 5 ASA + Ns | Remission | 2,74* | 1,35 | 5,59 | 0,00 |
|  | mild | 2,10* | 1,02 | 4,32 | 0,03 |
|  | moderate | 2,32* | 1,03 | 5,28 | 0,04 |
| Steroide + Immunomodulaors | Remission | 5,18* | 2,39 | 13,00 | 0,00 |
|  | mild | 2,67* | 1,22 | 6,76 | 0,01 |
|  | moderate | 1,62 | 0,67 | 4,41 | 0,28 |
| Biologica (Anti-TNFA-Alpha) + Ns | Remission | 0,50 | 0,11 | 1,40 | 0,21 |
|  | mild | 0,56 | 0,13 | 1,60 | 0,31 |
|  | moderate | 1,69 | 0,33 | 6,60 | 0,47 |
